# Water- and nitrogen-dependent alterations in the inheritance mode of transpiration efficiency in winter wheat at the leaf and whole-plant level

**DOI:** 10.1007/s13353-012-0107-z

**Published:** 2012-08-10

**Authors:** Dominika Ratajczak, Andrzej G. Górny

**Affiliations:** Institute of Plant Genetics, Polish Academy of Sciences, Strzeszyńska 34, 60-479 Poznań, Poland

**Keywords:** *Triticum aestivum* L., Adaptation, Combining ability, Drought, Inheritance, N shortage, Photosynthetic activity, Water use efficiency

## Abstract

The effects of contrasting water and nitrogen (N) supply on the observed inheritance mode of transpiration efficiency (TE) at the flag-leaf and whole-season levels were examined in winter wheat. Major components of the photosynthetic capacity of leaves and the season-integrated efficiency of water use in vegetative and grain mass formation were evaluated in parental lines of various origins and their diallel F_2_-hybrids grown in a factorial experiment under different moisture and N status of the soil. A broad genetic variation was mainly found for the season-long TE measures. The variation range in the leaf photosynthetic indices was usually narrow, but tended to slightly enhance under water and N shortage. Genotype–treatment interaction effects were significant for most characters. No consistency between the leaf- and season-long TE measures was observed. Preponderance of additivity-dependent variance was mainly identified for the season-integrated TE and leaf CO_2_ assimilation rate. Soil treatments exhibited considerable influence on the phenotypic expression of gene action for the residual leaf measures. The contribution of non-additive gene effects and degree of dominance tended to increase in water- and N-limited plants, especially for the leaf transpiration rate and stomatal conductance. The results indicate that promise exists to improve the season-integrated TE. However, selection for TE components should be prolonged for later hybrid generations to eliminate the masking of non-additive causes. Such evaluation among families grown under sub-optimal water and nitrogen supply seems to be the most promising strategy in winter wheat.

## Introduction

Water availability is the most critical environmental restriction to crop productivity. Its shortages are known to reduce wheat yields in many parts of the world, especially in arid/semi-arid areas. However, such stress conditions cause the same adverse effects in regions that are not commonly categorised to drought-prone habitats, but are recurrently identified to experience more or less severe water shortages at critical growth phases of the crop (Kijne et al. 2003; Fotyma 2004). In several European countries with permeable podzolic soils, periodic drought episodes and related disturbances in nutrient accessibility frequently cause substantial decreases and instabilities in wheat yield. In the central and west counties of Poland, for instance, the negative balance between evapotranspiration and precipitation is usually reported to be the major constraint to the grain production of winter wheat (Kędziora and Olejnik 2002; Łabędzki and Bąk 2005). Thus, the strategy to breed cultivars more efficient in water use and better adapted to less favourable water availability is justified and could stabilise wheat production in the region.

Among numerous morpho-physiological characteristics and mechanisms involved in the cereal response to water limitations, characteristics associated with rooting ability, photosynthetic capacity of plant foliage, stomatal functions, osmotic regulations, partitioning and secondary re-distribution of assimilates within plant organs appear to be crucial for the efficiency of water use and the complex plant performance under such conditions (Palta et al. 1994; van Ginkel et al. 1998; Richards et al. 2002; Reynolds 2002; Górny 2004; Slafer and Araus 2007; Yoo et al. 2009). In plants, water transpiration constitutes one of the central growth processes. Enhanced transpiration efficiency (TE), defined here at the plant level as dry matter of above-ground biomass produced per unit of water transpired during the whole growth season, appears to be an essential physiological attribute for maintaining soil moisture longer and a more satisfactory productivity in less favourable environmental habitats. Since photosynthetic functions of the uppermost leaves are essential during grain mass formation (Yang et al. 2000; Verma et al. 2004; Zhao et al. 2008), the instantaneous measure of the leaf efficiency of gas exchange, defined here at the flag-leaf level as the ratio of net CO_2_ assimilation to water transpiration (A/E), may be considered as an important leaf characteristic that contributes to the season-integrated TE. Furthermore, the appearance of a close relationship between carbon assimilation and leaf/plant N status may be crucial for TE and grain yield in environments with different soil N content (Evans 1989; Lawlor 2002).

According to the model proposed by Passioura (1977): Y = WT × TE × HI; the grain yield (Y) of water-limited plants depends upon the amount of water transpired (WT), plant capacity to an efficient use of the water in biomass formation (TE) and harvest index (HI). As already emphasised (Austin 1994), increases in yield potential of the recently released wheats are mainly attributed to enhanced HI, but these man-made alterations in the distribution of assimilates into grains appear to reach its physiological barrier. On the other hand, much less conscious efforts were made in wheat to improve the whole-season TE. Hence, further improvements in grain yield and its stability under variable water and nitrogen availability towards conscious breeding for increased TE and/or enhanced WT has become challenging (Richards et al. 2002; Slafer and Araus 2007; Sinclair 2012).

Although a broad genotypic variation in the season-long efficiency of water use was already reported and potential application of this knowledge for wheat improvement was suggested (e.g. Condon et al. 1990, 2004; Ehdaie 1995; Van den Boogaard 1995; Górny and Garczyński 2002; Tambussi et al. 2007), extremely few efforts have, hitherto, been made to breed wheat for the efficiency successfully. In essence, this was realised only by the researchers from the Australian Commonwealth Scientific and Industrial Research Organisation (CSIRO) (Rebetzke et al. 2002; Condon et al. 2004), who indirectly selected for carbon isotope discrimination (∆^13^C), i.e. a surrogate measure that, in C_3_ plants, usually exhibits a negative relationship with the season-long TE.

We have a reason to believe that technical questions associated with the precise, but labourious, recording of water transpired in extensive plant populations during their whole-growth season did not facilitate progress in such research activities. As a consequence, our understanding of the mode in which TE components are inherited is still limited in wheat and other cereals, and available data may lead one to not completely clear conclusions on the genetic scenario. Only few reports in bread and durum wheat deal with the inheritance of TE components at the leaf level, but less consistent or contrasting results have been reported for the CO_2_ assimilation rate, stomatal conductance and gas exchange efficiency (Carver et al. 1989; Simón 1994; Clarke 1997; Malik et al. 1999; Rebetzke et al. 2003). Nevertheless, the polygenic nature of the gas exchange efficiency, i.e. the instantaneous leaf TE measure at the seedling and generative growth phases and its complex genetic control, was recently substantiated by Zhang et al. (2005), Zhou et al. (2006) and Rebetzke et al. (2008) in their molecular studies on associated QTL regions.

On the other hand, extremely few efforts have been directed toward understanding the genetic basis for the seasonal TE, i.e. the trait measured on the whole-season-long scale. Nevertheless, the season-integrated TE exhibits quite high broad-sense heritabilities in wheat and barley (Ehdaie et al. 1993; Krzemińska and Górny 2003). In ditelosomics (Ehdaie and Waines 1997) and disomic substitutions (Górny 1999a, 2000), chromosomes from almost all wheat linkage groups were involved in the whole-season TE variation, suggesting multiple genes responsible for the complex plant character. The polygenic nature and relatively high narrow-sense heritabilities of the season-long TE were previously reported in the tetraploid durum wheat (Solomon and Labuschagne 2004) and spring barley (Górny 1999b). As far as conventional genetic/breeding approaches are concerned, no other data are available in the hexaploid wheat for the whole-season TE indices across either various levels of plant organisation or different environmental conditions. Since the limited knowledge may have an impact on the current breeding methodology and goals, more information on the manner in which TE components are inherited is rather necessary if benefits from an improved water use are to be realised in the species.

Thus, the major objectives of the study on winter wheat were: (i) to evaluate the inheritance mode of quantitative TE components and (ii) to identify effects of reduced N nutrition and low soil moisture on the phenotypic expression of genes responsible for TE at the leaf and whole-plant levels. For this purpose, hybrid progenies of crosses between lines representative for older and modern wheat germplasm of various origin were evaluated under contrasting water and N supply.

## Materials and methods

### Plant materials

All crosses excluding reciprocals were made in a diallel fashion among inbred lines (S_5_ to S_8_ of inbreeding) representative for the cultivars: Mironovska 808 (old extensive Ukrainian cv.; released in 1962; hereafter referred to as Mironovska), Maris Huntsman (old British cv., 1968), Korweta and Finezja (two modern Polish cvs., 2000/2002), and Clever and Kris (two modern, short-statured British cvs., 1999/2001) of winter wheat, *Triticum aestivum* L. The parental cultivars were previously identified to show distinct differences in yield potential/stability, rooting capacity, nutrient efficiency, photosynthetic activity of leaves and water use efficiency under varied water and nutrient supply. Native wheat breeders recognise them as donors of different qualitative and quantitative characteristics. After a reproduction of plant materials (Ps, F_1_s) under uniform conditions, parental lines and their F_2_ hybrids were investigated.

### Experimental design and data collection

The factorial experiment was arranged as a completely randomised design with 21 entries, three soil moisture/nutrition treatments, four pot-replications and 14 plants per pot. As detailed in a companion paper (Górny et al. 2011), plants were grown in double-walled Kick–Brauckmann’s experimental pots (9 dm^3^) in a partly controlled greenhouse. The pots were uniformly filled with a sieved soil mixture (loamy-sand soil:commercial peat, 1:2 by v/v, pH 6.4) of an optimal moisture (70 % of field water capacity), previously limed and mechanically mixed with water-soluted nutrients to achieve optimal (or initial for N) macro- and micronutrient contents. As monitored by a modified Spurway’s method, concentrations of plant-available macro- and microelements in the initial soil substrate were as follows: 80 N, 150 P, 250 K, 130 Mg (mg dm^−3^) and 85 Fe, 6 Zn, 4.5 Cu, 25 Mn and 0.8 B (ppm).

Eighteen uniform, surface-sterilised and pre-germinated seeds (filter paper, 24 h, 22 °C) were sown in each pot. After the first leaf emergence, 14 healthy seedlings were retained in each pot (density approximately 350 plants m^−2^) and the soil surface was covered with a 2.5-cm layer of perlite to eliminate soil water evaporation (EV). Check pots without plants were included to record the potential EV. The pot-grown juveniles were vernalised at 1–5 °C for 8 weeks. Later, 8–28/6–18 °C day/night mean temperatures, 10–18-h photoperiod and 50–85 % relative humidity (RH) were maintained/changed, depending on the growth stage and photoperiod cycling. To prolong the natural light duration during respective growth stages, an artificial lighting system was provided, supplying at least 400–800 μmol m^−2^ s^−1^ PAR at the plant level, as ensured by enhanced density (~1,100 W m^−2^) of high-pressure sodium and mercury lamps (both 400 W). To minimise the possible effects of light and temperature gradients, pots were frequently moved around the experimental area. Standard chemical treatments were used to control insects and pathogens.

The following three soil treatments were used: (1) Control: optimal soil moisture (70–75 % of the field water capacity) and N content in the soil, i.e. 235 mg N dm^−3^ soil; (2) Low N: optimal soil moisture and reduced N content in the soil, i.e. 140 mg N dm^−3^; and (3) Drought: optimal N content and reduced soil moisture, i.e. 40 % of the field capacity. In each treatment, nitrogen (as ammonium nitrate) was added in two water-soluted sub-portions: at the tillering stage (65 % of the whole rate) and before heading (35 % of the whole rate). In the third treatment, drought stress was attained by withholding watering for 2–3 days and the reduced water content (40 % FWC) was kept constant during the whole reproductive growth phase, beginning at the booting phase (4.2–4.4 Zadoks’ scale).

The amount of water transpired (WT) was recorded individually for each pot by its frequent weighing (2 to 7 times per week, depending upon the growth phase) and the required soil moisture (70–75 % or 40 % of FWC, depending upon the treatment) was maintained constant throughout the plant vegetation by the addition of respective volumes of deionised water into pots. At the most vigorous growth phase (heading–grain formation), pots were usually watered twice a day, i.e. in the early morning and late afternoon. The soil moisture was also monitored using the HH2 Wet Sensor (Delta-T, Cambridge, UK).

During the first week after heading (6.5–6.9 Zadoks’ scale), the photosynthetic rate (A; μmol m^−2^ s^−1^), transpiration rate (E; mmol m^−2^ s^−1^) and stomatal conductance (g_s_; mmol m^−2^ s^−1^) in the middle parts of fully expanded flag leaves were measured each day in the morning (9:00–11:30 a.m.) and afternoon (2:30–4:30 p.m.) using the portable infrared gas analyser LCA-4 (ADC Ltd., Hoddesdon, UK) that was equipped with the Parkinson’s leaf chamber (PLC-N) supplied with standardised air, drawn from outdoors 4 m above the ground. The leaf chamber was connected with a compatible PLU-2 light source (Tungsten 3,000°K) that provided a constant 1,100 μmol m^−2^ s^−1^ PAR energy at the measured leaf surface. Depending upon the recording time, pots were watered to the established soil water content in each evening of the preceding days (for forenoon readings) and in each morning (for afternoon readings). Temperature 24 ± 1°C, at least 600–700 μmol m^−2^ s^−1^ PAR at the plant level and 45–50 % RH were maintained during the five subsequent recording days. Altogether, four to five randomly chosen flag leaves were measured in each pot-replication during the five runs of readings done in a possibly randomised fashion. At least one leaf was measured per each experimental unit and running day. The instantaneous indices of the gas exchange efficiency, i.e. transpiration efficiency at the leaf level, were estimated as the photosynthesis/transpiration ratios (A/E).

Whole plants were hand-harvested at full maturity. The vegetative (stems + leaves) and generative (grains) plant parts were separated and their dry weights (g d.w. pot^−1^) were determined by oven drying at 65 °C for 72 h. The season-integrated vegetative (TE_VEG_) and generative (TE_GEN_) indices of transpiration efficiency (mg d.w. mol^−1^) were estimated by dividing vegetative and grain dry weights by moles of the whole-season WT (adjusted for EV), respectively.

### Data analysis

Standard statistical methods were used for data evaluations. The results of all measures in a replication were averaged and the analysis of variance (ANOVA) was performed on the basis of individual pot observations (MSTAT-C package, Michigan State University, 1990). Means were compared using Duncan’s multiple range tests. Using the SAS DGH v.1.0 package (Agricultural University of Poznań), variance components, genetic parameters and general and specific combining abilities (GCA and SCA, respectively) were estimated according to the Hayman’s, Griffing’s, and Mather and Jinks’ concepts for diallel mating, as detailed by Singh and Chaudhary (1985) and Kala et al. (1994).

## Results

### Variation in yielding capacity and water consumption

Generally, the hybrid progenies were able to produce grains and vegetative biomass with the same capacity as that compared with their parental forms (Table 1). The variation range in the amount of water transpired during the whole growth season in parents and hybrids was also similar. Some transgressive effects were noticed only in the harvest index. Under low N nutrition, the entries consumed about 13 % less water than in control conditions. On an average, their vegetative dry weight was similarly reduced (by 14.3 %), while their grain dry weight decreased only by 10.7 %, and this effect was accompanied with a significant enhancement in the translocation of dry matter into grains. The used drought stress caused distinctly greater depressions in the characteristics. Though the amount of water used decreased by about 28 %, the dry weights of grains and vegetative parts were found to be depressed by 22.6 and 19.4 % only, suggesting that some compensative effects likely associated with a stress-induced increase in the efficiency of water use.

**Table 1:** Variation range (minimal and maximal means) among parental and hybrid generations of winter wheat and average soil treatment effects for the grain dry weight (GEN), vegetative dry weight (VEG), harvest index (HI) and total amount of transpired water (WT)

Variation source	GEN, g d.w.	VEG, g d.w.	HI, %	WT, dm^3^
Entries				
Parents	31.1–41.8	31.7–48.2	43.2–50.6	22.8–26.1
F_2_ hybrids	29.8–42.2	31.2–49.5	43.1–52.2	22.4–26.4
LSD_0.05_	2.3	1.8	1.5	1.0
Soil treatments				
Control	39.3	44.9	46.8	28.3
Low N	35.1	38.5	47.8	24.6
Drought	30.4	36.2	45.6	20.4
LSD_0.05_	0.9	0.7	0.6	0.4

### Variation in transpiration efficiency and its associations with grain yield

As summarised in Table 2, parental lines and their hybrid progenies exhibited considerable differences in components of TE at either the leaf or whole-plant level. In general, the F_2_ means for most characters were within the range of parents. However, flag leaves of some hybrid progenies tended to exchange CO_2_/H_2_O with at least identical or higher leaf gas exchange efficiency (A/E) than those of their best parents, indicating positive transgressive effects in the leaf photosynthetic efficiency. The effects were usually associated with equivalent decreases in transpiration rate (E) and stomatal conductance (g_S_).

**Table 2:** Variation range (general means) among parental and hybrid generations, average soil treatment effects, results of the pooled analysis of variance (mean squares) and broad-sense heritabilities (h_BS_^2^) for components of TE at the leaf and whole-plant level in winter wheat

Variation source		Flag leaves				Whole plants	
		A	E	g_S_	A/E	TE_VEG_	TE_GEN_
		μmol m^−2^ s^−1^	mmol m^−2^ s^−1^	mmol m^−2^ s^−1^	mmol mol^−1^	mg mol^−1^	mg mol^−1^
Entries							
Mironovska (Mir)		16.0	2.25	154	7.19	34.5	29.9
Maris Huntsman (MHu)		17.1	2.36	164	7.31	31.3	23.8
Clever (Cle)		13.8	2.11	143	6.59	24.9	25.5
Kris (Kri)		17.4	2.17	150	8.09	24.6	24.5
Finezja (Fin)		16.1	2.08	159	7.73	31.8	24.9
Korweta (Kor)		17.1	2.26	148	7.69	27.7	23.7
F_2_ (Mir × MHu)		16.5	2.09	150	8.00	33.9	28.9
F_2_ (Mir × Cle)		15.7	2.01	142	7.90	30.4	28.0
F_2_ (Mir × Kri)		16.2	1.91	138	8.59	29.6	27.8
F_2_ (Mir × Fin)		17.5	2.15	154	8.34	33.9	28.6
F_2_ (Mir × Kor)		17.3	2.09	157	8.48	31.9	27.1
F_2_ (MHu × Cle)		14.7	1.87	133	7.98	29.2	26.5
F_2_ (MHu × Kri)		15.4	1.94	146	8.02	28.4	25.1
F_2_ (MHu × Fin)		15.7	2.01	143	7.91	32.1	27.0
F_2_ (MHu × Kor)		16.2	2.05	145	8.05	30.3	22.8
F_2_ (Cle × Kri)		15.1	2.00	136	7.68	24.4	26.5
F_2_ (Cle × Fin)		15.1	2.02	136	7.52	29.4	24.0
F_2_ (Cle × Kor)		15.7	1.92	139	8.38	27.1	23.5
F_2_ (Kri × Fin)		16.8	1.86	140	9.26	27.5	25.5
F_2_ (Kri × Kor)		16.6	1.90	145	8.93	26.2	23.6
F_2_ (Fin × Kor)		16.9	1.99	153	8.64	30.0	24.3
LSD_0.05_		1.2	0.18	13	0.74	1.2	1.3
Soil treatments							
Control		17.2	2.20	156	7.94	28.4	24.9
Low N		15.5	1.94	143	8.06	28.1	25.7
Drought		15.7	2.01	140	8.04	31.9	26.8
LSD_0.05_		0.4	0.07	5	ns	0.4	0.5
ANOVA	(d.f.)						
Treatments (T)	2	70.5**	1.53**	5790**	0.35	364.7**	71.0**
Entries (E)	20	11.2**	0.22**	821**	4.41**	109.8**	49.4**
T × E	40	4.8**	0.09*	522**	1.07	2.5	7.4**
Error	189	2.0	0.05	273	0.82	2.2	2.2
Heritability, h_BS_^2^		0.51	0.47	0.34	0.57	0.94	0.80

*A* photosynthetic rate; *E* transpiration rate; *g*_*S*_ stomatal conductance; *A/E* leaf gas exchange efficiency; *TE*_*VEG*_ transpiration efficiency in vegetative mass formation; *TE*_*GEN*_ transpiration efficiency in grain mass formation

*, ** - significant at the *P* = 0.05 and *P* = 0.01 levels, respectively

In general, the magnitude of the genotypic variation in leaf photosynthetic indices was only small or moderate (Table 2). The pooled broad-sense heritabilities (h_BS_^2^) ranged from 0.57–0.47 for A/E, A and E to 0.34 for g_S_, indicating stronger influences of environmental forces on the instantaneous leaf characteristics than on the whole-season TE measures. In the examined wheat collection, the season-long TE indices exhibited a distinctly broader genetic variation than the leaf TE components, as heritabilities of the former attained at least 0.80. Interestingly, the range of genotypic differences for the transpiration efficiency in vegetative mass formation (TE_VEG_) was wider than that for TE in grain mass formation (TE_GEN_), resembling, rather, the fact that variability found in Ps and F_2_s in vegetative vigour was distinctly broader than that in their grain weight (Table 1).

Except for the leaf A/E, all characters were significantly affected by soil treatments (Table 2). Generally, leaf photosynthetic measures decreased under stress conditions, while the season-long TE efficiencies tended to increase, especially under drought conditions. However, the results of the pooled analysis of variance indicated that the magnitude of the stress-induced alterations in most characters was dependent on the genotype, as evidenced by the significant entry × treatment interactions. Such interactive effects were not only found for A/E and TE_VEG_.

There was a weak or non-significant relationship between the grain weight and photosynthetic features of the flag leaf (Table 3). Some positive associations between A, g_S_ and E and yielding capacity were only found in entries grown under control conditions. Similarly, no correlation between HI and GEN was observed. By contrast, GEN was closely associated with the season-integrated components of the transpiration efficiency under all soil treatments, especially with WT and TE_GEN_. These strong and positive relationships indicate that at least 77–85 % of variation in yielding capacity was determined by the efficiency of water use in grain mass formation. Hence, the potential importance of the integrated TE for selection dealing with superior yielding was corroborated.

**Table 3:** Phenotypic correlations between grain yield (GEN; g dry weight) and examined components of transpiration efficiency (TE) in winter wheat under different soil treatments

	GEN (d.w.)		
	Control	Low N	Drought
A, photosynthetic rate	0.58**	−0.21	0.09
E, transpiration rate	0.40	−0.27	0.13
g_S_, stomatal conductance	0.44*	−0.28	0.19
A/E, gas exchange efficiency	0.01	−0.02	−0.05
HI, harvest index	0.12	0.13	0.33
WT, water transpiration	0.79**	0.55**	0.64**
TE_VEG_, TE in vegetative mass formation	0.63**	0.59**	0.52*
TE_GEN_, TE in grain mass formation	0.90**	0.92**	0.88**

*, ** - significant at the *P* = 0.05 and *P* = 0.01 levels, respectively

### Combining ability effects

ANOVA for combining abilities was performed separately for each treatment to identify possible influences of soil treatments on the genotypic variation and relative importance of general and specific combining ability (GCA and SCA, respectively) effects. As presented in Table 4, the season-long TE indices were found to exhibit the widest genotypic variation. The range of this variation in most photosynthetic leaf features was usually smaller; however, the extent of variation in A and A/E of leaves slightly increased under stress conditions.

**Table 4:** Analysis of variance (mean squares), relative proportion of GCA-dependent variance (GCA ratio), genotypic coefficient of variation (G.C.V.) and the relationships between the performance of parents and their GCAs (r Y_*r*_-GCA) for the leaf and whole-plant components of TE in winter wheat under different soil treatments

Character^a^	Soil treatment	Variation source				GCA ratio^b^	G.C.V. (%)	r Y_*r*_-GCA
		Entries	GCA	SCA	Error			
A	Control	5.60**	15.47**	2.31	2.41	0.93	5.2	0.96**
	Low N	7.23**	11.80**	5.70**	2.22	0.81	7.2	0.94**
	Drought	7.99**	21.88**	3.35**	1.36	0.93	8.2	0.85*
E	Control	0.189**	0.431**	0.109**	0.044	0.89	8.7	0.98**
	Low N	0.052	0.041	0.055	0.038	n^c^	n^c^	n^c^
	Drought	0.149**	0.080	0.172**	0.056	0.48	7.9	0.73
g_S_	Control	697**	1,724**	355	263	0.91	6.7	0.95**
	Low N	436*	386	452*	230	0.63	5.0	0.93**
	Drought	732**	1,036**	631**	262	0.77	7.7	0.64
A/E	Control	1.60*	1.17	1.75*	0.85	0.57	5.4	0.85*
	Low N	2.20**	3.38**	1.81*	0.74	0.79	7.5	0.87*
	Drought	2.75**	5.25**	1.91*	0.97	0.85	8.3	0.87*
TE_VEG_	Control	36.6**	141.8**	1.6	1.9	0.99	10.5	0.98**
	Low N	43.6**	162.8**	3.9*	2.0	0.99	11.4	0.98**
	Drought	35.0**	132.3**	2.5	2.4	0.99	8.9	0.98**
TE_GEN_	Control	17.9**	57.1**	4.8**	2.0	0.96	8.0	0.97**
	Low N	20.7**	56.4**	8.7**	1.5	0.93	8.5	0.92**
	Drought	25.9**	66.9**	12.2**	2.2	0.92	9.1	0.84*

^a^Trait abbreviations as in Table 1

^b^Calculated according to Baker (1978) as 2MS_GCA_/(2MS_GCA_ + MS_SCA_)

^c^Not estimated due to a lack of genetic variation

*, ** - significant at the *P* = 0.05 and *P* = 0.01 levels, respectively

All the alterations were accompanied with changes in the relative contribution of GCA and SCA effects (Table 4). If not exclusively, the former effects were found to be highly significant and dominating causes (GCA ratios ≥ 0.8) for the variation in the season-long TE indices and A in each treatment, for E and g_S_ in the control plants and for A/E under both stress conditions. In turn, SCA-dependent effects were identified as sole and critical causes of variation in E under drought, g_S_ under N shortage and A/E in control plants. Consequently, the importance of the GCA-dependent influences on the total variance tended to decline, as indicated by reduced GCA ratios (0.48–0.63) for these traits. As far as the season-long TE indices are concerned, SCA effects were significant for the vegetative TE_VEG_ under low N nutrition only, whereas their importance for the generative TE_GEN_ indices was found to increase under water and nitrogen shortages, suggesting enhanced expression of non-additive gene action in such conditions.

The GCA effects of parental lines are presented in Table 5. In general, a regularity of the effects across experimental treatments was found, except for some leaf characteristics. The cv. Clever tended to reduce A and g_S_, while the cvs. Mironovska and Korweta exhibited a tendency to increase the characters in their hybrid progenies, especially under drought stress. The relatively weak consistency of GCAs for the leaf characteristics was indicative for GCA–treatment interactions that were mainly associated with the magnitude of GCA effects. However, progenies of the cvs. Kris and Mironovska exhibited more critical interactions, as substantial changes in the sign of GCAs were detected for A and g_S_, respectively.

**Table 5:** General combining abilities of parental wheat lines for the leaf and whole-plant TE components under different soil treatments

Character^a^	Soil treatment	Parents					
		Mironovska 808	Maris Huntsman	Clever	Kris	Finezja	Korweta
A	Control	0.84**	0.07	−1.00**	−0.63*	0.18	0.54*
	Low N	−0.45	−0.04	−0.77**	0.96**	−0.04	0.35
	Drought	0.48*	−0.10	−1.62**	0.37	0.32	0.55**
E	Control	0.12**	0.08*	−0.11**	−0.17**	0.02	0.07
	Low N	−0.05	0.02	0.04	0.01	−0.03	0.02
	Drought	0.08*	0.03	−0.04	0.01	−0.04	−0.04
g_S_	Control	9.48**	0.13	−10.29**	−6.62*	4.95	2.34
	Low N	−6.30*	2.94	0.44	−1.28	1.32	2.89
	Drought	5.72*	4.61	−10.14**	0.85	0.83	−1.87
A/E	Control	−0.04	−0.23	−0.11	0.33*	0.00	0.05
	Low N	0.00	−0.15	−0.54**	0.43**	0.15	0.11
	Drought	−0.11	−0.19	−0.65**	0.21	0.24	0.50**
TE_VEG_	Control	2.57**	1.33**	−2.01**	−2.80**	1.24**	−0.35
	Low N	3.01**	1.42**	−2.18**	−2.81**	1.17**	−0.60*
	Drought	2.77**	1.08**	−1.88**	−2.29**	1.41**	−1.09**
TE_GEN_	Control	2.47**	−0.25	−0.39	−0.44	0.15	−1.54**
	Low N	1.97**	−0.31	0.17	−0.03	0.36	−2.16**
	Drought	2.90**	−0.41	−0.15	−0.65**	−0.97**	−0.72**

^a^Trait abbreviations as in Table 1

*, ** - significant at the *P* = 0.05 and *P* = 0.01 levels, respectively

As shown in Table 4, a general consistency between parental means and GCAs was observed under all experimental conditions for most characters, especially for the seasonal TEs and the instantaneous A and A/E leaf indices. The largest positive GCA effects for the season-long TE measures were exhibited by the most water-efficient Ukrainian cv. Mironovska, while the inefficient Polish cv. Korweta contributed towards reduced TEs in its progenies. In turn, the both short-statured and less efficient British cvs. Clever and Kris appeared to be the last combiners for the efficiency of water use in vegetative mass formation (TE_VEG_).

### Genetic components of variation

Estimates of genetic components of variation for the examined characters are presented in Table 6. For some leaf characteristics under optimal conditions (i.e. A and A/E), however, this estimation was unreasonable because of the failure of adequacy of the additive-dominance model (Wr–Vr regression slope ≠ 1) for A or limited parental variance being smaller than the error variance in A/E. The presented data corroborate that additive gene action (D) was significant for all characters studied, except for E and g_S_ under N shortage. If significant, H estimates were positive, indicating that the dominance of increasing alleles was significant for the characters examined. The contribution of H_1_ was frequently larger than H_2_, suggesting that positive and negative alleles at loci responsible for most characters were unequally distributed in parents, and this was especially apparent in stress conditions. A further proof for the less symmetrical allele distribution in parents was also provided by the quantities H_2_/4 H_1_ ≠0.25.

**Table 6:** Estimates of genetic components of variance (D, F, H_1_, H_2_, h_2_), average degree of dominance [(0.25 H_1_/D)^1/2^] and narrow-sense heritabilities (h_NS_^2^) for the leaf and whole-plant TE components in winter wheat under different soil treatments

Character^a^	Soil treatment	Genetic components					(0.25 H_1_/D)^1/2^	h_NS_^2^
		D	F	H_1_	H_2_	h_2_		
A	Control	n^b^	n^b^	n^b^	n^b^	n^b^	n^b^	n^b^
	Low N	3.98**	5.29**	3.81**	2.06*	0.12	0.49	0.80
	Drought	1.74**	0.10	1.98*	1.14*	0.94*	0.53	0.44
E	Control	0.048**	0.006	0.031	0.036*	0.155**	0.40	0.38
	Low N	0.003	0.002	0.006	0.009	0.052**	0.72	n^d^
	Drought	0.022*	0.040*	0.077*	0.057*	0.220**	0.94	0.15
g_S_	Control	181**	31	60	70	126	0.29	0.26
	Low N	17	−20	141	169	245**	1.44	0.05
	Drought	131*	177*	333*	187	372**	0.80	0.22
A/E	Control	n^c^	n^c^	n^c^	n^c^	n^c^	n^c^	n^c^
	Low N	0.69**	0.73**	1.17**	0.88**	0.74**	0.65	0.51
	Drought	0.40*	−0.01	0.53	0.46	2.16**	0.57	0.25
TE_VEG_	Control	13.86**	−0.85**	0.61	0.55	0.03	0.11	0.84
	Low N	14.01**	−3.76**	0.50	0.62	7.20**	0.09	0.78
	Drought	12.80**	−0.80*	1.35*	0.89	0.26	0.16	0.80
TE_GEN_	Control	4.44**	−1.67**	1.75	1.66*	4.50**	0.31	0.55
	Low N	6.19**	1.54**	6.51**	5.30**	0.76	0.51	0.71
	Drought	7.29**	3.43**	10.16**	6.57**	0.08	0.59	0.68

^a^Trait abbreviations as in Table 1

^b, c, d^Not estimated because of the inadequacy of the additive-dominance model, error variance greater than the variance of parents or a lack of genetic variation, respectively

*, ** - significant at the *P* = 0.05 and *P* = 0.01 levels, respectively

As revealed by the h_2_/H_2_ proportions, at least 2–11 sets of genes exhibiting dominance were found to govern all the TE_VEG_ and TE_GEN_ in control plants, E and g_S_ under stress, as well as A/E under water shortage. Noteworthy, the contribution of the gene groups for the season-integrated TE_GEN_ appeared to decline in stress conditions, resulting in enhanced narrow-sense heritabilities.

The covariance of additive and dominance effects is measured by the *F* component. If significant, the *F* estimates were mainly positive, suggesting a preponderance of dominant alleles responsible for the leaf photosynthetic capacity in parents. However, the sign of the *F* estimate for the season-long TE_GEN_ was found to vary between soil treatments being negative under control conditions or positive under stress, thus, indicating some inequality of gene frequencies with an excess of recessive or dominant alleles governing the character in optimal conditions or in water- and N-deprived plants, respectively. Interestingly, a higher frequency of dominant alleles controlling water vapour exchange of leaves (i.e. E and g_S_) under drought conditions was identified to coincide with that for the integrated TE_GEN_. Similar consistency of gene frequencies was noticed between the leaf A and A/E and the season-long TE_GEN_ in plants grown under N shortage. In all conditions, however, an excess of recessive alleles governing TE_VEG_ in parents was found.

The average degree of dominance, (0.25 H1/D)^1/2^, was usually within a range of partial dominance (degree < 1). Overdominance (degree > 1) was only noticed for the genetically less variable stomatal conductance (g_S_) under low N nutrition, while almost complete dominance was found for E and g_S_ during drought stress. Noteworthy, the degree of dominance involved in the inheritance of most characters was low under optimal conditions, but tended to enhance under nitrogen and water shortages.

## Discussion

In the present study on the hexaploid winter wheat, significant genetic variation in components of transpiration efficiency at the flag-leaf and whole-plant level was identified. The variation was evident for the TE components under all soil treatments, except for the transpiration rate of flag leaves in control plants. The magnitude of the genotypic differences in the seasonal transpiration efficiency in vegetative mass formation (TE_VEG_) was greater than that for TE in grain mass formation (TE_GEN_), suggesting stronger environmental influences on all succeeding morpho-physiological processes/mechanisms decisive for the spike formation, grain quantity and grain filling, i.e. for components of grain yield structure. Nevertheless, the variation in the season-integrated TE measures was nearly 2- to 3-fold broader than that in leaf photosynthetic capacity, corroborating both a variable nature of the later and limited extent of its genetic variability available among actually collected wheats (Carver et al. 1989; Simón 1994; Górny et al. 2006). The results suggest that a much broader genetic variation among the regional wheat germplasm should be expected in the season-integrated TEs than in the instantaneous TE indices at the flag-leaf scale. Since the leaf efficiency could be particularly important at the reproductive growth phase during which the uppermost leaves serve as essential sources of plant assimilates for developing grains (Verma et al. 2004; Zhao et al. 2008), the results of the study raise a principal query as to whether the magnitude of the genetic variation in leaf TE components is sufficiently wide among modern wheat collections to substantiate their use in breeding process. The results, rather, imply that searching for a novel variation in the photosynthetic activity of the uppermost leaves is justified if a progress in breeding winter wheats with improved photosynthetic performance is to be realised.

The used soil treatments were found to exhibit considerable influence on the phenotypic expression of gene action responsible for the examined TE components. Interestingly, though negligible or only moderate range of the additivity-dependent variation was found for some flag-leaf and whole-plant indices (A, A/E and TE_GEN_) under optimal conditions, the magnitude of this variation, as measured by narrow-sense heritabilities (h_NS_^2^), tended to increase among nitrogen- and/or water-deprived entries, indicating a stress-induced expression of additive gene action. As far as the season-long TE is concerned, the results fit well within the drought-induced heritability increases of TE_GEN_ described by Solomon and Labuschagne (2004) in cross-progenies of tetraploid durum wheats of the Ethiopian collection. On the other hand, our observation appears to be in a conflict with the high heritabilities of the photosynthetic rate and transpiration efficiency of the 4th leaves identified by Malik et al. (1999) in juvenile spring wheat under drought conditions. Noteworthy, the authors examined cross-progenies of Pakistan and British spring wheats previously chosen for likely separate genetic factors governing their ABA-dependent stomatal functions and specific adaptation to drought-prone habitats.

However, it should be pointed that the materials examined in our study appear to be representative for a European gene pool different from that described by Malik et al. (1999). Firstly, the magnitude of the additive variation in the leaf TE components found in the present study was distinctly restricted as compared with that in the spring wheat reported by Malik and co-workers. Secondly, the stress-induced enhancement of additivity-dependent variance in A and A/E was more evident under low N nutrition than under drought conditions. Hence, the examined parents of European origin seem to signify a wheat collection with uppermost leaves that exhibit dissimilar reactions to such environmental limitations. Namely, the old and modern parents appear to consist of a much broader pool of additive genes governing a response of their photosynthetic apparatus to different N nutrition levels than that of factors associated with the response of photosynthetic machinery to water shortage. Considering the close relationship between the plant/leaf N status and photosynthesis (Kumar et al. 2002; Lawlor 2002), this variation seems to have its origin in the breeding history of the parental cultivars and, likely, different selection forces acting during their development under dissimilar N fertilisation regimes used in the various agricultural eras and countries of their release.

In general, the results of the study in winter wheat indicate that additive gene action was more pronounced in the inheritance of most TE components. However, the dominance of genes was also involved in the control of the examined characters. Hence, possible conclusions on the preponderance of additive gene action, which could arise from the higher proportions of GCA over SCA effects, e.g. for the leaf photosynthetic rate and the season-integrated TEs, did not hold true for the transpiration rate and stomatal conductance under drought and low N nutrition. Namely, the proportion of the GCA-dependent variance of the last traits tended to decrease under stress conditions, while the average degree of dominance increased. It is difficult to identify factual genetic grounds for the stress-induced attenuation of additivity-dependent variation, but a possible protective role of the dominance gene action for stomatal functions in sub-optimal environments should not be neglected.

Nevertheless, inferences to be drawn on the manner of gene action responsible for TE components are dependent on the answer as to whether the additive-dominance model of our analysis was valid to explain the observed variation. The present analysis was carried out with a supposition that at least major Hayman’s assumptions for an adequacy (or a partial adequacy) of the additive-dominance model (i.e. homozygous parents, diploid segregation, no maternal/reciprocal effects, no epistasis, no linkage, no multiple alleles, no lethal genes etc.) were satisfied. The winter wheat is a hexaploid crop, but its chromosomes behave as in a diploid species and diploid segregation is assured. There is a predominantly self-pollinated species and circa 95 % of self-pollination is natural here. The parental lines chosen for the study were obtained after several generations of inbreeding, so the parents were expected to be at least 97/98 % homozygous. Furthermore, adequacy of the additive-dominance model was also tested using the statistic the DGH package and this was confirmed for almost all characters, except for the photosynthetic rate (A) in optimal conditions, for which the model was inadequate because of non-allelic interaction (epistasis) and/or independence of genes among parents.

The remaining assumptions on multiple alleles and a lack of linkage and reciprocal effects could only be partly verified following an analysis of complete diallel or other mating systems. Since a half diallel was evaluated in our study, there was no opportunity to identify maternal and non-maternal reciprocal causes. This appears to be an important point, especially when leaf photosynthetic parameters are discussed. Namely, the involvement of both nuclear and cytoplasmic genes in the control of carbon metabolism is well known in plants. Given that not only nuclear genes, but also the extra-nuclear, i.e. chloroplastic, genes are important for the photosynthetic capacity, we must believe that, in our analysis, only a partially adequate model was still used. Therefore, some caution is necessary when interpreting our data on the leaf photosynthesis. Undoubtedly, since the maternal/cytoplasmic effects could be adaptive, further knowledge of how the cytoplasmic and nuclear factors interact to modulate leaf photosynthesis under diverse environmental habitats may be valuable in this wheat collection.

It is commonly believed that genetic modifications of the photosynthetic process in wheat offer novel chances for further improvement of the species. However, our results did not confirm the potential for fast genetic modifications of the photosynthetic TE efficiency of flag leaves in winter wheat under diverse environmental habitats. The restricted extent of additivity-dependent variation actually available among modern wheats implies that the use of leaf photosynthetic parameters in selection process may be limited, and search for novel variation in the photosynthetic performance of the uppermost leaves is justified if progress in breeding winter wheats with improved photosynthetic efficiency during the reproductive growth phase is appreciated. Furthermore, strong caution and precision are necessary during the photosynthetic readings, and to reduce experimental error, i.e. to enhance the operative heritability of a trait, such measures should likely be undertaken in possibly numerous experimental repetitions/replications. Hence, methodical troubles may occur in practice for even well-equipped research teams during a respective growth phase when replicated and usually repeated photosynthetic readings among large plant populations during a limited time of run-days are needed.

In conclusion, the results of the study did not provide evidence to indicate that measures of the leaf photosynthetic activity and photosynthetic efficiency will be convenient criteria in wheat selection. As far as the season-integrated TE indices are concerned, a broader genetic variation and more favourable inheritance mode found for the TEs indicate that more promise exists in European winter wheat for the improvement of its water use efficiency via conventional breeding methods. A consistency between parental performance per se and GCAs was noticed for major TE indices. As pointed by Le Gouis et al. (2002) at the occasion of their genetic study on N efficiency in wheat, such consistency appears to be especially desirable for the breeding practice showing an opportunity to envisage the hybrid efficiency on the basis of parental values. However, such conventional and likely plain approach to improve TE_GEN_, i.e. to enhance the efficiency of water use in grain mass formation on the basis of selection for GCA effects, may be less effective with the realisation that non-additive gene action could significantly mask the variation, especially in early hybrid generations of wheats grown under sub-optimal water and nitrogen supply. The data provide evidence that searching for improved TE_GEN_ may be less successful when conducted under optimal conditions only. Parallel evaluation of TE components in plants grown under sub-optimal water and N supply should additionally be considered by wheat researchers dealing with improved adaptation of the hexaploid wheat to less favourable water and N availability in the environment. Furthermore, substantial contribution of non-additive gene action for TE suggests that such evaluation of progenies should be prolonged for later hybrid generations to eliminate the masking non-additive causes. Hence, this may be a labour-intensive and time-consuming task, even for well-organised teams, when handling extensive cross-progenies would be necessary. Alternatively, use of the earlier-mentioned surrogate TE measure, i.e. carbon isotope discrimination (Rebetzke et al. 2002; Condon et al. 2004) or selective categorisation of such wide plant materials with the assistance of molecular markers (Zhou et al. 2006; Rebetzke et al. 2008) may be projected.

## References

[CR1] Austin RB, Boote KJ (1994). Plant breeding opportunities. Physiology and determination of crop yield.

[CR2] Baker RJ (1978). Issues in diallel analysis. Crop Sci.

[CR3] Carver BF, Johnson RC, Rayburn AL (1989). Genetic analysis of photosynthetic variation in hexaploid and tetraploid wheat and their interspecific hybrids. Photosynth Res.

[CR4] Clarke JM (1997). Inheritance of stomatal conductance in a durum wheat cross. Can J Plant Sci.

[CR5] Condon AG, Farquhar GD, Richards RA (1990). Genotypic variation in carbon isotope discrimination and transpiration efficiency in wheat. Leaf gas exchange and whole plant studies. Aust J Plant Physiol.

[CR6] Condon AG, Richards RA, Rebetzke GJ, Farquhar GD (2004). Breeding for high water use efficiency. J Exp Bot.

[CR7] Ehdaie B (1995). Variation in water use efficiency and its components in wheat. II. Pot and field experiments. Crop Sci.

[CR8] Ehdaie B, Waines JG (1997). Chromosomal location of genes influencing plant characters and evapotranspiration efficiency in bread wheat. Euphytica.

[CR9] Ehdaie B, Barnhart D, Waines JG, Ehleringer JR, Hall AE, Farquhar GD (1993). Genetic analysis of transpiration efficiency, carbon isotope discrimination and growth characters in bread wheat. Stable isotopes and plant carbon-water relations.

[CR10] Evans JR (1989). Photosynthesis and nitrogen relationships in leaves of C_3_ plants. Oecologia.

[CR11] Fotyma M (2004) Gateway to land and water information: Poland national report. http://www.apipnm.org/swlwpnr/reports/y_te/z_pl/pl.htm

[CR12] Górny AG (1999). Effects of D-genome substitutions on the water use efficiency and response of the ‘Langdon’ durum wheat (*Triticum turgidum* L. var. *durum*) to reduced nitrogen nutrition. Cereal Research Comm.

[CR13] Górny AG (1999). Inheritance of water use efficiency in diallel hybrids of spring barley under varied nutrition and soil moisture. J Appl Genet.

[CR14] Górny AG (2000). Effect of the substituted A and B chromosomes of *Triticum dicoccoides* on the nitrogen, phosphorus and water use efficiency in the ‘Langdon’ durum wheat (*T. turgidum* L. var. *durum*). Cereal Res Comm.

[CR15] Górny AG (2004). Genetics and physiology of cereal roots in response to water and nutrient shortages. Z Probl Post Nauk Roln.

[CR16] Górny AG, Garczyński S (2002). Genotypic and nutrition-dependent variation in water use efficiency and photosynthetic activity of leaves in winter wheat (*Triticum aestivum* L.). J Appl Genet.

[CR17] Górny AG, Garczyński S, Banaszak Z, Ługowska B (2006). Genetic variation in the efficiency of nitrogen utilization and photosynthetic activity of flag leaves among the old and modern germplasm of winter wheat. J Appl Genet.

[CR18] Górny AG, Banaszak Z, Ługowska B, Ratajczak D (2011). Inheritance of the efficiency of nitrogen uptake and utilization in winter wheat (*Triticum aestivum* L.) under diverse nutrition levels. Euphytica.

[CR19] Kala R, Chudzik H, Dobek A, Kiełczewska H (1994) SAS DGH v.1.0: Statistical Analysis System for Genetic and Breeding Experiments. Agricultural University of Poznań

[CR20] Kędziora A, Olejnik J, Ryszkowski L (2002). Water balance in agricultural landscape and options for its management by change in plant cover structure of landscape. Landscape ecology in agroecosystems management.

[CR21] Kijne JW, Barker R, Molden D (2003). Water productivity in agriculture: Limits and opportunities for improvement.

[CR22] Krzemińska A, Górny AG (2003). Genotype-dependent variation in the transpiration efficiency of plants and photosynthetic activity of flag leaves in spring barley under varied nutrition. J Appl Genet.

[CR23] Kumar PA, Parry MAJ, Mitchell RAC, Ahmad A, Abrol YP, Foyer CH, Noctor G (2002). Photosynthesis and nitrogen-use efficiency. Photosynthetic nitrogen assimilation and associated carbon and respiratory metabolism.

[CR24] Łabędzki L, Bąk B (2005) Drought mapping in Poland using SPI. In: Proceedings of the 21st ICID Conference, Frankfurt, Germany, 15–19 May 2005, pp 1–4

[CR25] Lawlor DW (2002). Carbon and nitrogen assimilation in relation to yield: mechanisms are the key to understanding production systems. J Exp Bot.

[CR26] Le Gouis J, Béghin D, Heumez E, Pluchard P (2002). Diallel analysis of winter wheat at two nitrogen levels. Crop Sci.

[CR27] Malik TA, Wright D, Virk DS (1999). Inheritance of net photosynthesis and transpiration efficiency in spring wheat, *Triticum aestivum* L., under drought. Plant Breed.

[CR28] Palta JA, Kobata T, Turner NC, Fillery IR (1994). Remobilization of carbon and nitrogen in wheat as influenced by post-anthesis water deficits. Crop Sci.

[CR29] Passioura JB (1977). Grain yield, harvest index and water use of wheat. J Aust Inst Agric Sci.

[CR30] Rebetzke GJ, Condon AG, Richards RA, Farquhar GD (2002). Selection for reduced carbon-isotope discrimination increases aerial biomass and grain yield of rain-fed bread wheat. Crop Sci.

[CR31] Rebetzke GJ, Condon AG, Richards RA, Farquhar GD (2003). Gene action for leaf conductance in three wheat crosses. Aust J Agric Res.

[CR32] Rebetzke GJ, Condon AG, Farquhar GD, Appels R, Richards RA (2008). Quantitative trait loci for carbon isotope discrimination are repeatable across environments and wheat mapping populations. Theor Appl Genet.

[CR33] Reynolds MP, Curtis BC, Rajaram S, Gómez Macpherson H (2002). Physiological approaches to wheat breeding. Bread wheat: improvement and production.

[CR34] Richards RA, Rebetzke GJ, Condon AG, van Herwaarden AF (2002). Breeding opportunities for increasing the efficiency of water use and crop yield in temperate cereals. Crop Sci.

[CR35] Simón MR (1994). Gene action and heritability for photosynthetic activity in two wheat crosses. Euphytica.

[CR36] Sinclair TR (2012). Is transpiration efficiency a viable plant trait in breeding for crop improvement?. Funct Plant Biol.

[CR37] Singh RK, Chaudhary BD (1985). Biometrical methods in quantitative genetic analysis.

[CR38] Slafer GA, Araus JL, Spiertz JHJ, Struik PC, van Laar HH (2007). Physiological traits for improving wheat yield under a wide range of environments. Scale and complexity in plant systems research. Wageningen UR Frontis Series, vol 21.

[CR39] Solomon KF, Labuschagne MT (2004). Inheritance of evapotranspiration and transpiration efficiencies in diallel F_1_ hybrids of durum wheat (*Triticum turgidum* L. var. *durum*). Euphytica.

[CR40] Tambussi EA, Bort J, Araus JL (2007). Water use efficiency in C_3_ cereals under Mediterranean conditions: a review of some physiological aspects. OPTIONS Méditerranéennes Ser B.

[CR41] Van den Boogaard R (1995) Variation among wheat cultivars in efficiency of water use and growth parameters. PhD thesis, Faculty of Biology, Utrecht University, pp 1–155

[CR42] Van Ginkel M, Calhoun DS, Gebeyehu G, Miranda A, Tian-you C, Pargas Lara R, Trethowan RM, Sayre K, Crossa J, Rajaram S (1998). Plant traits related to yield of wheat in early, late, or continuous drought conditions. Euphytica.

[CR43] Verma V, Foulkes MJ, Worland AJ, Sylvester-Bradley R, Caligari PDS, Snape JW (2004). Mapping quantitative trait loci for flag leaf senescence as a yield determinant in winter wheat under optimal and drought-stressed environments. Euphytica.

[CR44] Yang JC, Zhang JH, Huang ZL, Zhu QS, Wang L (2000). Remobilization of carbon reserves is improved by controlled soil-drying during grain filling of wheat. Crop Sci.

[CR45] Yoo CY, Pence HE, Hasegawa PM, Mickelbart MV (2009). Regulation of transpiration to improve crop water use. Crit Rev Plant Sci.

[CR46] Zhang J, Zhang ZB, Xie HM, Dong BD, Hu MY, Xu P (2005). Chromosomal positioning of the genes of water use efficiency and concerned physiological traits in wheat leaves. Acta Bot Boreal-Occident Sin.

[CR47] Zhao H, Zhang ZB, Shao HB, Xu P, Foulkes MJ (2008). Genetic correlation and path analysis of transpiration efficiency for wheat flag leaves. Environ Exp Bot.

[CR48] Zhou X-G, Jing R-L, Chang X-P, Zhang Z-B (2006). QTL mapping for water use efficiency and related traits in wheat seedling. J Plant Genet Resour.

